# Repurposing mucosal delivery devices for live attenuated tuberculosis vaccines

**DOI:** 10.3389/fimmu.2023.1159084

**Published:** 2023-03-30

**Authors:** Munish Puri, Socorro Miranda-Hernandez, Selvakumar Subbian, Andreas Kupz

**Affiliations:** ^1^ College of Public Health, Medical and Veterinary Sciences, James Cook University, Townsville, QLD, Australia; ^2^ Centre for Molecular Therapeutics, Australian Institute of Tropical Health and Medicine, James Cook University, Cairns, QLD, Australia; ^3^ Public Health Research Institute (PHRI), New Jersey Medical School, Rutgers University, Newark, NJ, United States

**Keywords:** tuberculosis, vaccine delivery, Respimat^®^ Soft Mist^™^, mucosal atomization device (MAD) syringe, mucosal vaccine, mucosal

## Abstract

Tuberculosis (TB) remains one of the most lethal infectious diseases globally. The only TB vaccine approved by the World Health Organization, Bacille Calmette-Guérin (BCG), protects children against severe and disseminated TB but provides limited protection against pulmonary TB in adults. Although several vaccine candidates have been developed to prevent TB and are undergoing preclinical and clinical testing, BCG remains the gold standard. Currently, BCG is administered as an intradermal injection, particularly in TB endemic countries. However, mounting evidence from experimental animal and human studies indicates that delivering BCG directly into the lungs provides enhanced immune responses and greater protection against TB. Inhalation therapy using handheld delivery devices is used for some diseases and allows the delivery of drugs or vaccines directly into the human respiratory tract. Whether this mode of delivery could also be applicable for live attenuated bacterial vaccines such as BCG or other TB vaccine candidates remains unknown. Here we discuss how two existing inhalation devices, the mucosal atomization device (MAD) syringe, used for influenza vaccines, and the Respimat^®^ Soft Mist^™^ inhaler, used for chronic obstructive pulmonary disease (COPD) therapy, could be repurposed for mucosal delivery of live attenuated TB vaccines. We also outline the challenges and outstanding research questions that will require further investigations to ensure usefulness of respiratory delivery devices that are cost-effective and accessible to lower- and middle-income TB endemic countries.

## 1 Introduction

Lower respiratory tract infections, excluding COVID-19, are the fourth major cause of death worldwide, with an annual incidence rate of 2.4 million (1). More than half of the mortality associated with respiratory infections is due to tuberculosis (TB), a bacterial disease predominantly caused by infections with *Mycobacterium tuberculosis* (Mtb). Although TB affects people globally, it is most prominent in low- and middle-income countries. In 2021, 10.6 million people suffered from active TB and 1.6 million people lost their lives worldwide (2).

Bacille Calmette-Guérin (BCG) is the only vaccine approved by the World Health Organization (WHO) to prevent disseminated forms of TB (meningitis and miliary) in children (3). BCG is a live attenuated strain of *Mycobacterium bovis*, a genetically related mycobacterial species to Mtb. The vaccine provides variable protection in adults, with the longest-lasting protection of 50-60 years reported in Alaskan natives and American Indians (4) and of 10-19 years in a Norwegian population (5). It has also been reported that BCG is less efficacious in TB endemic countries, including Asia and Africa, compared to non-endemic countries (6). The potential reasons for this variability in BCG efficacy have been discussed elsewhere (7).

BCG is marketed as a freeze-dried powder in an amber glass ampoule and transported to vaccination centers (8). Just before vaccination, the vial is reconstituted with a diluent (provided in a separate vial) at the vaccination center, and is administered to newborns (and sometimes older children) within six hours of reconstitution *via* the intradermal route by trained personnel (9). The BCG vaccination process can be daunting for both the receiving individual and the administering health worker.

There is mounting evidence from animal model studies (10–17) and historical observations in humans (18–24) that delivery of BCG *via* the mucosal route may be more efficacious and provides longer-lasting protection than the conventional intradermal route. While the correlates of protection after BCG vaccination are poorly defined/unknown, enhanced protection after mucosal vaccination has been linked to the generation of tissue-resident T cells (T_RM_). While intranasal, oral, intravenous and intratracheal route of BCG vaccination induces T_RM_ in the lungs, parenteral intradermal or subcutaneous vaccination fails to do so (14, 16, 25–38).

Hence, many research groups have supported and proposed pulmonary delivery of TB vaccines, and progress has been made recently in this area of research (39). It is important to note that in addition to developing (a) suitable TB vaccine candidate(s) for mucosal delivery, investigations into suitable delivery devices for mass human application should be prioritized. As a disease of poverty in resource-limited and populous countries (40), mass vaccination using a reusable aerosol delivery device attached to a medical breathing circuit appears unviable and may carry a high risk of unintended TB transmission. Therefore, we believe that research efforts should be directed toward developing and evaluating a simple, cost-effective, user-friendly, and easily available single-use device with the ability to deliver the vaccine deep into human lungs. Both BCG (41) and MTBVAC (a promising TB vaccine in clinical trials) delivery *via* the intranasal route has successfully been tested in animal models, including those with co-morbidities (42). However, there is no published information on what kind of delivery device could deliver a live attenuated TB vaccine intranasally or intratracheally into human lungs. Below we discuss some of the challenges and opportunities associated with the mucosal delivery of live attenuated TB vaccines into human lungs.

## 2 Factors associated with vaccine efficacy by mucosal delivery

Generally, the efficacy and immunogenicity of a vaccine rely on a range of factors associated with the host, the environment, and the vaccine formulation, including the type, dose, administration route, needle size (if applicable), co-administered vaccines and timing (6). Vaccination with a live, attenuated BCG was more immunogenic than inactivated bacteria (43, 44), and the liquid formulation provoked a stronger immune response than the powder formulation (45). These results suggest that microbial viability in the vaccine formulation influences the host immune response, and that the ideal BCG replacement vaccine should constitute live bacteria.

Depending on the nature of the infecting Mtb strain, a minimum of three tubercle bacilli are enough to establish a productive infection (46). Inhalation of Mtb-containing aerosol droplets facilitates tubercle bacilli to reach the lung parenchyma, where they first encounter the alveolar lining fluid (ALF) or pulmonary surfactant. This fluid is comprised of lipids (90%) and proteins (10%). The lipid components decrease the surface tension and alter the multiplication and function of lymphocytes (47). On the other hand, the proteins interact with the surface glycolipids of the tubercle bacilli (48). Overall, the ALF actuates the pathogen capture and clearance by phagocytic cells such as alveolar macrophages. However, the absence of ALF due to preexisting conditions such as asthma or COPD increases host susceptibility to various infections, including TB (46, 49–51). It has been reported that Mtb blocks phago-lysosome fusion by perturbing the pH of the phagosome to 6.4. This suggests that Mtb prefers living in a slightly acidic environment, which may have important implications for the choice of diluent used to reconstitute the vaccine, to maximise the stability and viability of mucosally delivered BCG.

The Mtb/*M. bovis* cell wall consists of a thick waxy coat that protect the bacteria from the outside environment, contributing to the bacterial resistance to antibiotics (52). Based on pulmonary Mtb infection in model animals, it seems that this waxy coat contributes to bacterial survival in host lungs with varying physiological (pH, surface, partial pressure of oxygen and carbon dioxide, relative humidity, temperature and density) and microbial gradients (53). Thus, the waxy coat of live mycobacterial strains can impact the delivery of the vaccine directly into the lungs and affect subsequent host immune responses.

Despite lacking the region of difference 1 (RD1) (7), BCG contains several ‘decoy’ molecules, such as the glycoprotein LprG and Lipoarabinomannan (LAM), which evolved in support of virulence of mycobacteria. Both molecules delay the protective Th1 immune response *via* the induction of immunosuppressive cytokines and chemokines and could hence be counterproductive to prophylactic vaccination (54, 55).

## 3 Likelihood of mucosal vaccination in humans

One of the biggest challenges in TB vaccine development is the lack of a human challenge model that could be used to evaluate the efficacy of new vaccine candidates. This is not surprising as it is not possible to safely infect humans with Mtb due to ethical reasons. However, the TB research group at Oxford University has recently started recruiting a small number of healthy human volunteers (aged 18 to 50) to participate in a BCG challenge trial. This study assesses whether humans can be safely infected with BCG *via* the aerosol route. In the trial, volunteers receive an escalating dose of BCG using an aerosol delivery device. Subsequently, the researchers intend to collect lung washings of the volunteers to determine the amount of recoverable BCG, to discover new biomarkers potentially involved in protection against TB, and to validate the BCG challenge model as a new way to test TB vaccine candidates (56). Although the primary aim of this study is not to test the efficacy of mucosal BCG delivery as a vaccination strategy against subsequent Mtb infection, the trial will likely provide critical information regarding the safety and tolerability of aerosol BCG vaccination. In addition, this trial will likely help determine an optimal BCG dose for mucosal vaccination to minimize any BCG dose-dependent lung pathology reported by Tree et al. in a murine model of TB (57).

In fact, the respiratory tract as a potential vaccine delivery route is under active investigation for other infections. For the last two decades, several research groups have proposed the pulmonary site as an ideal target to deliver vaccines and induce immunity to combat respiratory infections (58–61). To date, there are only six licensed intranasal vaccines for humans (**Table 1**), including vaccines against COVID-19 (68). This delay in the development of inhaled vaccines highlights the challenges underpinning the delivery of antigens into the respiratory tract. However, given the promising results from animal studies, an inhaled vaccine may be what is needed to protect against TB.

**Table 1:** Licensed mucosal vaccines for human use.

Vaccine	Origin	Route	Effective against	Reference
Flumist	USA	IN	Influenza	(62)
Nasovac	INDIA	IN	Influenza	(63)
iNCOVACC	INDIA	IN	COVID-19	(64)
Convidecia Air™	CHINA	IT	COVID-19	(65)
Razi Cov Pars	IRAN	IM/IN	COVID-19	(66)
Sputnik V	RUSSIA	IN	COVID-19	(67)

IN, intranasal; IM, Intramuscular and IT, intratracheal.

## 4 Potential TB vaccine delivery devices

A key component in mucosal delivery of a live TB vaccine to millions of people globally, including children, is the inhalation device. There are more than 230 inhalers available with varying mechanics that could conceptually be used to deliver TB vaccines (**Table 2**) (69, 72). For example, COPD and asthmatic patients are routinely prescribed specific inhalers for delivering disease-specific drugs directly into the lungs. This raises the possibility of using these pulmonary inhalers to deliver live mycobacteria vaccines. Currently, a broad range of liquid, mist, and powder-based inhalers are available and frequently prescribed (73). Based on the vaccine (liquid or powder) formulation, some of those available inhalers could be validated as a delivery device for inhaled live attenuated TB vaccines. However, given the low cost of current intradermal BCG vaccination, the cost of goods associated with making an inhalation delivery device will need to be considered to allow for global rollout. Furthermore, a needle-free delivery system, as an alternate to the current intradermal BCG vaccination should be simple and easy to use in infants and young children, particularly in resource-limited TB endemic countries.

**Table 2:** Summary of available inhaler types and suitability for TB vaccine delivery (69–72).

Inhaler type	Strengths	Weaknesses
Metered dose Inhalers (MDI)	* Compact size * User friendly * Most treatment options available in this format * Reproducible dose * Drug delivery is independent of inhalation flow * Suitable for emergencies	o Use propellants for better drug delivery o Device priming required o Hand-lung coordination required o Variable and inconsistent dose delivery on not properly shaking the device prior to use
Dry powder inhalers	* Small size * An alternative to propellent-based inhalers * Available as a single and multi-dose system	o Dependent on the patient’s inspiration flow rate and inhaler turbulence o Sensitive to humidity requiring specific storage conditions o Powder o The drug-containing capsule needs to be loaded into the device
Soft Mist Inhalers	* Portable and user friendly * Independent of the patient’s inspiration flow * High fine particle fraction leads to deep lung deposition	o The drug-containing cartridge needs to be loaded into the inhaler o Device priming required o Delivers only limited medication types (no corticosteroids)
Nebulizers	* Applicable to small children, disabled and elderly patients	o Large size o Require regular proper cleaning o Prone to deliver respiratory viral infections o Device performance is highly variable o Substantial particle deposition on nasal mucosa, eyes, and skin (on using nose-mouth masks) o Requires external sources of power and compressed gas o Substantial drug leftovers in the reservoir in some devices after use o Not recommended for long-term asthma and COPD treatment

In general, the inhalation capacity is compromised in those with preexisting health conditions such as asthma or COPD. In such situations, it is important to choose an inhaler whose actuation mechanism is preferably independent of the user’s inspiration rate to ensure the delivery of an accurate vaccine dose. This criterion narrows the list of suitable inhalers to only a few (**Table 2**). Below we elaborate on one potential device each for intratracheal and intranasal inhalation.

### 4.1 Intratracheal

**Figure 1:**
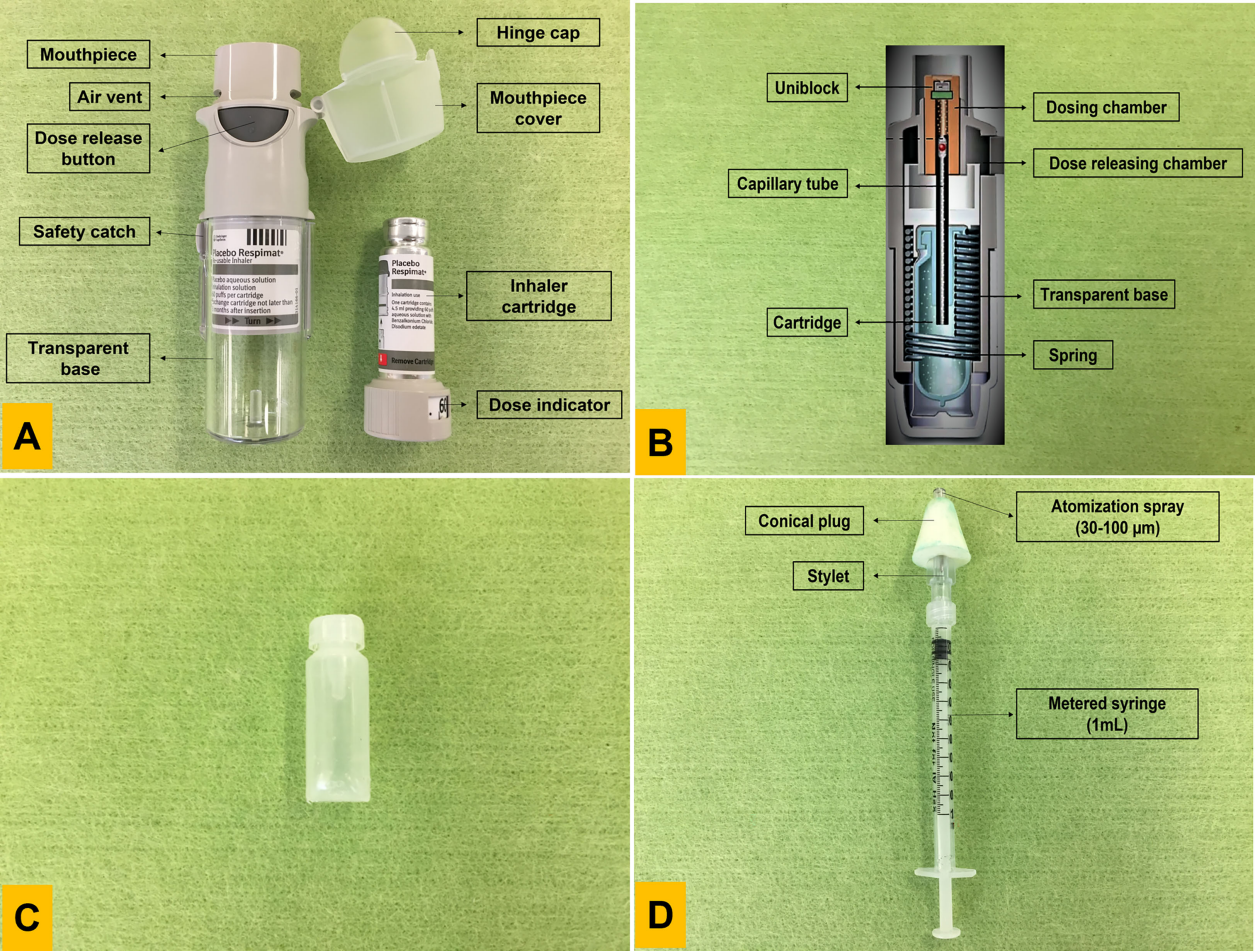
The Respimat inhaler and Mucosal Atomization Device syringe. **(A)** Usability diagram explaining its structural components and cartridge; **(B)** Cross section of the inhaler vertically to understand the working mechanics of the device; **(C)** Respimat inhaler cartridge that holds the drug. The plastic pouch is protected by an outer aluminium shell connected to a dose indicator; **(D)** The atomization device is connected to the syringe for mist production. Both components can be separated and reconnected as a single unit. Photo B was modified from (74).

The Respimat^®^ Soft Mist™ inhaler (SMI; Boehringer Ingelheim, Ingelheim, Germany) (**Figure 1A**) is prescribed as an inhalation therapy to patients with COPD. It is a multidose, propellent-free liquid inhaler that produces an aerosol plume (75) of small droplet size with low momentum (76) on pressing a button. It delivers the bronchodilators (10-15 µL per actuation) at the velocity of 0.8m/sec that last longer (1.5 seconds) than other propellant-based inhalers. This allows synchronization of actuation and inspiration for better drug delivery (77).

A spring (**Figure 1B**) provides mechanical energy that forces the solution through a fine nozzle (uniblock), thereby atomizing the solution release as two fine liquid jets. When those jets collide at a pre-set angle, it causes the production of a particle cloud or soft mist, which allows inhalation deep into the lung (78). A study by Taube et al. analysed data from two independent studies, comprised of over 90,000 patients on the performance of Respimat^®^ Soft Mist™ inhaler. They reported that 85% and 84% of patients, respectively, were content with using and handling the inhaler. Interestingly, over 95% of people continued using the inhaler after the study ended (79).

Could the Respimat^®^ Soft Mist™ inhaler be used as a mucosal TB vaccine delivery device? Conceptually, replacing the solution contained in the Respimat^®^ Soft Mist™ inhaler cartridge with the live TB vaccine may be possible. The inhaler cartridge (**Figure 1C**) is composed of an aluminium cylinder that accommodates a contractable double-walled plastic pouch containing liquid solution (80). However, open questions such as the following remain: 1) Can the cartridge deliver viable mycobacterial bacilli deep into the lungs? 2) Can the device deliver a consistent dose? 3) What is the viability of live mycobacteria within the cartridge environment? 4) Is the nozzle size suitable for delivery of the waxy cell wall of mycobacteria? 5) Is the inhaler suitable for use in infants and young children? 6) What are the implications for storage, transport, and shelf life of the inhaler?

### 4.2 Intranasal

The mucosal atomization device (MAD) syringe (**Figure 1D**) is composed of a metered syringe (usually 1mL) connected to an atomization device that generates aerosols similar to the Respimat^®^ Soft Mist™ inhaler. The MAD consists of an atomization spray (the mist generator), an inlet to allow a 180° posture of a nasal plug, and a soft conical plug that acts as a nose seal to abstain expulsion of the vaccine. The mist produced by the device generates particles of 30-100 µm size. The device is patient actuated. The syringe plunger pushes the liquid through the MAD, and the atomization device converts the pushed liquid into a mist.

The device mechanism is simple and has successfully been used to deliver influenza vaccines such as Flumist™ and Nasovac. The simplistic aerosol-producing mechanism of the syringe makes the MAD economical and affordable. Furthermore, the convenience, ease of use and availability in low- and middle-income countries imply that the MAD could be another potential delivery device for live TB vaccines. Given that the MAD has already been approved and used to deliver an influenza vaccine *via* the intranasal route (**Table 1**), regulatory approval processes and manufacture should be translatable to TB vaccines.

Recently, Wei et al. have reported that 0.3mL was the optimum volume for intranasal delivery of drugs *via* the MAD without tracheal aspiration in rabbits. However, with a delivery volume of 0.45 or 0.6mL, the MAD was able to deliver particles to the rabbit trachea (81). Although these findings demonstrate a proof-of-principle that the MAD syringe can potentially deliver drugs/vaccines to the lower respiratory tract, further investigations are required to evaluate the optimal delivery volume of TB vaccines, particularly in humans.

BCG is generally administered right after birth or within the first few months of life (7). Compared to adults, newborns are characterized by distinctive breathing patterns (high breathing frequency up to 40 breaths per minute) (82). This variation is due to the difference in the nasal anatomy, chest wall geometry, respiratory muscles, presence of fluids and pressure (83). Wilkins and colleagues evaluated the MAD syringe for its intranasal vaccine delivery using five nasal replicas of infants aged 3-24 months. The study reported an overall delivery efficacy of 86.57 ± 14.23% for all models when administering 0.1mL of a model vaccine in each nostril (84). While these findings suggest that the MAD syringe may be suitable as a TB vaccine delivery device for neonates, the Respimat^®^ Soft Mist™ inhaler on the other hand may require further evaluations as a suitable delivery device for newborns. Nevertheless, based on the mechanics described above, it is likely that both delivery devices could be used to deliver not only BCG but also TB vaccine candidates that are based on viral vectors, protein subunits or nucleic acids.

## 5 Discussion

To further validate the suitability of the Respimat^®^ Soft Mist™ inhaler and the MAD for the delivery of live mycobacteria, the following points should be considered: firstly, the devices should be evaluated for consistency in the delivery dose. Once accurate delivery has been confirmed, testing could be extended to 3D models of the human respiratory system. Using a human respiratory CT scan, specialized software such as Mimics (Materialise, Belgium) could print a 3D human respiratory system using a medical-grade printer. This would allow inhaler efficiency testing in a human-like anatomical system. Lastly, the proposed devices should be tested using a next-gen impactor, a specialized pharmaceutical instrument used to determine the drug/vaccine’s aerodynamic particle size and depth of deposition of particles in the lungs.

**Figure 2:**
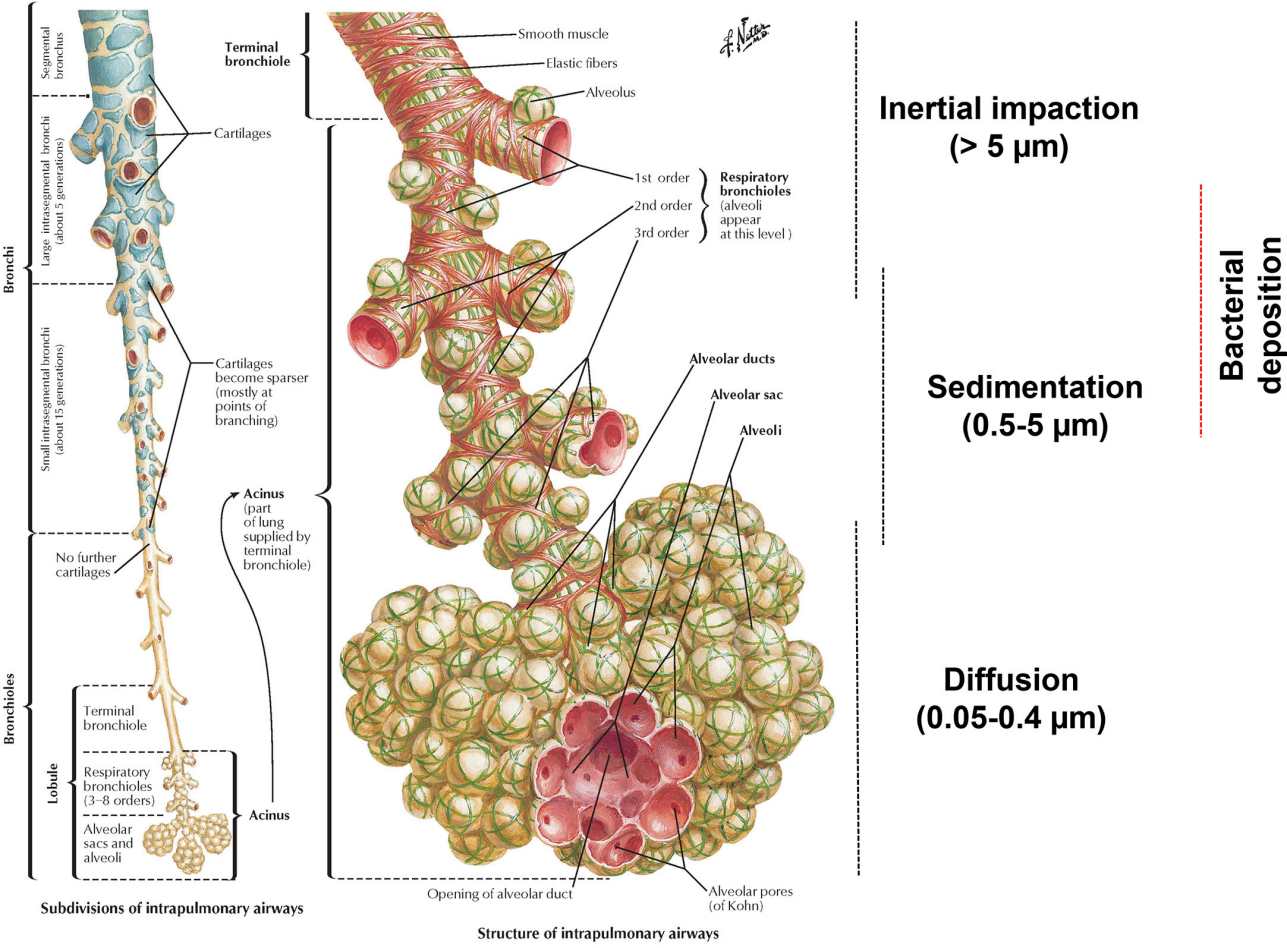
The relationship between particle size and lung deposition. The left section demonstrates the structure of intrapulmonary airways. The right section details the bacterial deposition based on size (0.5-10 µm) in the lower respiratory tract (86). The picture usage rights were purchased from netterimages.com (Image ID: 49221).

The rod-shaped *M. bovis* is 2-4 µm long and 0.2-0.5 µm in diameter (85). This size is compatible with the minimum requirement of a particle (0.5-5 µm aerodynamic size) to get delivered deep into the lungs by sedimentation (**Figure 2**) (87). Adding a surfactant to the liquid vaccine formulation would further decrease the surface tension and increase the vaccine dispersal inside the lungs (88). Nevertheless, maintaining the vaccine formulation within the device without a reduction in bacterial viability represents another challenge. However, based on the literature from BCG vaccine studies, it is logical to assume that cold storage of cartridges containing the vaccine formulations should be feasible without significantly compromising the bacterial viability. The widespread global use of mRNA-based COVID-19 vaccines, which require storage at very low temperatures, has shown that logistics and storage conditions are no longer a bottleneck for maintaining and delivering vaccines that require deep freezing.

Currently, BCG is given predominantly to children through intradermal injection, and as such mucosal delivery devices for BCG vaccination should be applicable to the national vaccination program for children of all age groups, including infants in TB-endemic countries. In addition, BCG re-vaccination in adolescents and adults is receiving renewed interest for improving the protection against TB. In addition to the unexpected positive results of the BCG re-vaccination control group in an H4IC31 trial (89), Rakshit et al. demonstrated that BCG re-vaccination (TUBERVAC, BCG Russia) greatly stimulated Ag85A and BCG-specific CD4^+^ T and CD8^+^ T cells in previously BCG vaccinated (at birth) health care workers. Additionally, BCG re-vaccination also increased mycobacteria-specific Th 17 responses (90), and a systematic review conducted by Bannister et al. indicated that BCG is safe to use as a booster dose (91). Collectively, these studies demonstrate the benefits of a booster BCG dose in adolescents and adults. Thus, an easy-to-use mucosal delivery device would likely simplify BCG re-vaccination programs.

The licensed BCG vaccine strains are available as a lyophilized powder (1.5 mg) that is reconstituted with a diluent (0.9% sodium chloride and diluted Sauton medium) just before administration (92). However, the proportion of viable bacteria in the prepared vaccine suspension strongly influences the magnitude of the host immune response (8, 45). It is also known that the thermal stability of a powder formulation is greater than that of a liquid (93). Hence, it is feasible to assume that the cartridges for mucosal delivery devices could also be filled with lyophilized bacteria, with the ability to reconstitute at the vaccination center just before inhalation.

The user instructions of approved mucosal delivery units strongly recommend holding the breath for at least 10 seconds immediately after usage (94, 95). This is followed by rinsing the mouth with water in some cases. Therefore, while delivering a live attenuated recombinant TB vaccine with these types of inhalers or an intranasal syringe may require additional procedures to ensure accidental exposure and/or transmission of the vaccine to other people or the environment. All these issues require further investigation, and data from such studies need to be considered when designing a vaccine delivery device for TB control.

Currently, the Respimat^®^ Soft Mist™ inhaler costs approximately US$45, excluding the cartridge. It is a multi-dose inhaler that holds 4.5 mL of fluid in each cartridge. The same inhaler can be used for up to six cartridges before being discarded. However, a more simplistic version could be developed to deliver a single-dose TB vaccine. For example, by excluding the mouthpiece cap and dose indicator (**Figure 1A**), a more transparent base could be produced to verify proper cartridge insertion. Such a generic, single-use and easily understandable mechanism might assist in reducing the overall manufacturing cost of the delivery device. Local production may also impact manufacturing costs and may increase affordability in low- and middle-income countries.

Since the Respimat^®^ Soft Mist™ inhaler delivers the content of the cartridge independently of the patient’s inhalation rate (96), it could also be used to deliver a TB vaccine effectively in patients with co-morbid respiratory diseases. The optical density of the bronchodilator solution given for COPD using the Respimat^®^ Soft Mist™ inhaler will likely differ from the TB vaccine candidate formulation. This may require further adjustments to the device (angle to produce fine liquid jets, increasing nozzle diameter, compatibility of the plastic cartridge pouch with the vaccine etc.). The MAD syringe, on the other hand, costs around US$6 and is based on relatively simple mechanics. If proven efficient in delivering a live attenuated TB vaccine *via* the pulmonary route, this device may have broad application globally, including in resource-limited countries.

In conclusion, emerging research findings support the idea that mucosal delivery of TB vaccines might confer superior protection than intradermal inoculations. Here, we propose that mucosal delivery of live attenuated TB vaccines, including BCG, is feasible and could be done by repurposing existing delivery devices for other respiratory diseases. Further research is required to dissect the challenges outlined above and to validate the usability of various inhaler types. Once proven technically feasible and safe, introducing an inhalation platform for the TB vaccine will undoubtedly contribute to increased vaccination rates globally and, consequently, to a reduction of TB burden.

## Data availability statement

The original contributions presented in the study are included in the article/supplementary material. Further inquiries can be directed to the corresponding author.

## Author contributions

MP wrote the manuscript and proposed the delivery devices. AK, SM-H, and SS provided editorial and intellectual input. All authors contributed to the article and approved the submitted version.
